# A case of methanol poisoning leading to prolonged respirator dependency with consequent blindness and irreversible brain damage 

**Published:** 2015

**Authors:** Ali Asghar Manuchehri, Ebrahim Alijanpour, Mohsen Daghmechi, Naser Ghaeminan, Seyed Hasan Abedi, Novin Nikbakhsh, Seyed Ali Mohammad Ghazi Mir Saeed, Neda Amani

**Affiliations:** 1 Department of Internal Medicine, Babol University of Medical Sciences, Babol, Iran.; 2 Department of Anesthesiology, Babol University of Medical Sciences, Babol, Iran.; 3 Department of Radiology, Babol University of Medical Sciences, Babol, Iran.; 4 Department of Surgery, Babol University of Medical Sciences, Babol, Iran.; 5 Clinical Research Development Center Shahid Beheshti Hospital Babol University of Medical Sciences, Babol, Iran.

**Keywords:** Methanol intoxication, Vision loss, Brain hemorrhage, Mechanical ventilation

## Abstract

**Background:**

Methanol is a colorless and poisonous liquid that is commonly used as an industrial and household solvent. Methanol poisoning is a rare but extremely hazardous form of in toxication that affects the central nervous system and causes visual disorder, drowsiness, seizures and coma.

**Case Presentation:**

A 29-years-old man with methanol intoxication presented with drowsiness and acidosis with subsequent brain hemorrhage requiring prolong mechanical ventilation resulted in persistent visual impairment and disequilibrium.

**Conclusion:**

Prolonged mechanical ventilation in patients with methanol intoxication may be assouated with irreversible brain damage.

**M**ethanol (wood alcohol) is a colorless, flammable and poisonous liquid that is commonly used as an industrial solvent (1).Poisoning with methanol may occur following accidental or purposeful ingestion of industrial solutions containing methanol such as antifreeze or ingestion of out of standard homemade alcohol. Methanol intoxication is extremely lethal and even moderate ingestion of this substance for suicidal purpose or as alcoholic drink substitution may be associated with major neurological complications such as drowsiness, seizures, coma and visual loss (2). Concomitant ingestion of methanol with ethanol may be associated with delayed onset of clinical symptoms as well as delayed initiation of treatment. Patients with larger amount of methanol may present with more severe central nervous system (CNS) complications involving different portions of CNS as well as visual system (3). In patients with brain injury requiring intubation and mechanical ventilation for longer duration and extubation and weaning from ventilator may be associated with difficulties (4-6). Prolonged duration of hospitalization increases the risk of nosocomial pneumonia (7) and affects disease course and outcome. 

## Case presentation

A 29-year-old man was admitted to emergency room 30 hours after ingestion of about 1 liter of homemade alcohol. The clinical picture began with frequent vomiting, blurred vision and back pain. Initial examination demonstrated an agitated and confused patient with respiratory distress. The respiratory rate was 26 per minute, pulse rate of 82 per minute, blood pressure of 130/80 mm Hg and axillary temperature of 36.5 C and laboratory tests as presented in table 1.

**Table 1:** The results of the laboratory tests admition

BS: 180	BUN: 18	Na: 134	WBC: 13600
Pco_2_: 38.2	Cr: 1.6	K: 5.3	RBC: 5200
Po_2_: 62	Urea: 52	Cl: 98	Hb: 15.8
Hco_3_: 9.5	ALT: 15	Ca:10.1	HCT: 49.6
O_2_ sat: 85.4	AST: 22	AG: 26.5	MCV: 85.8
BE: -20.8	ALK: 196	OG: 132	PLT: 231000

In clinical examination both pupils were dilated and the response to light was slow. The results of examination of the heart, lungs were normal and the findings of chest x-ray were unremarkable. There was no cyanosis or edema.

The patient was intubated in emergency room and mechanical ventilation was started. The PH was 7.017, gavage of ethanol, infusion of sodium bicarbonate was started and folic acid, pantoprazole was administered in accordance with intravenous fluids. In quantitative test of methanol assessment, the serum methanol level was estimated at the level of 50 mg/dl. The victim underwent two courses of hemodialysis. Over the course of poisoning, the level of Glasgow Coma Scale (GCS) deteriorated and on the seventh admission day the status of GCS remained at the level of B-C. The pupils were not reactive. The patients was required to be under ventilator in intensive care unit (ICU). The results of the laboratory tests at this time are presented in table 2. 

**Table 2:** The results of the laboratory tests in ICU

LDH: 555	Total PRO: 5.6	RBC: 3.6
Cr: 9	Ca: 8.9	Hb: 9.9
BIL T: 8	Mg: 1.4	HCT: 30.1
BIL D: 2	AST: 68	PT: 13
CPK: 435	ALK: 270	PTT: 36
INR: 1	ALT: 110	ALB: 3.4

**Figs. 1, 2:**
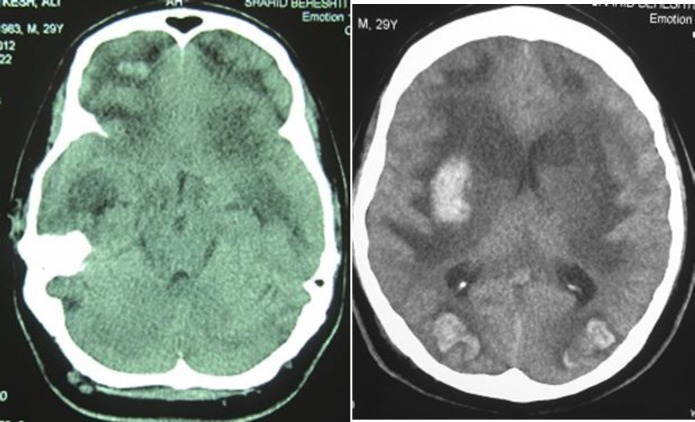
A: Large and symmetric edema in parenchymal of brain, foci hematoma intraparenchymal, subcortical frontal and occipital lobe bilaterally. B: Hematoma in putamen and right capsule

Two units of packed cells were transfused and a brain CT scanning showed large and symmetric brain edema as well as brain hematoma in subcortical brain over frontal and occipital lobes bilaterally as well as in putamen and right capsule areas. (figs 1, 2). Repeated clinical examinations, x-rays of the heart and lungs as well as the results of laboratory tests were normal and several attempts of weaning patients from the ventilator were unsuccessful and so a tracheostomy as well as percutaneous endoscopic gastrostomy was performed on the 18th day of hospitalization. Combined enteral and parenteral nutrition was continued according to current treatment criteria (6). The status of consciousness improved over the hospitalization period and the patient could be detached from the ventilator on the 79th day of hospitalization with GCS level of 9-10.

**Figs 3, 4:**
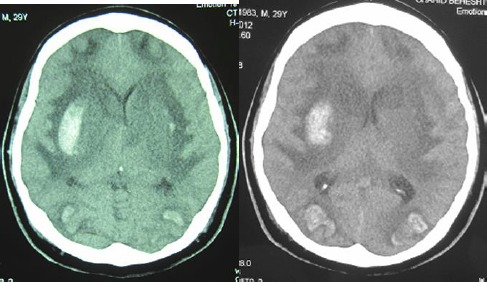
A: B: parenchymal atrophy of the right and left frontal lobes symmetrically, bilateral hypo density in the external capsule, left internal capsule and bilateral caudate and lateral ventricular dilatation were also seen

Follow-up examinations were continued for six months. At the latest examination the general condition of patient improved a little as compared during hospitalization time and was poor because of visual and neurological complications. The patient had urinary and fecal incontinence. The visual capacity of patient was limited to recognizing only a shaking hand and the potential of motor system was limited to slight movement of the hands and feet. The latest brain CT scanning demonstrated symmetrical brain atrophy of both frontal lobes. The lateral ventricles were dilate d, there were hypodensities in external capsule, left internal capsule and bilateral caudate areas (Figs 3, 4).

## Discussion

This study indicates the outcome of methanol toxicity following ingestion of large amount of homemade alcohol with resultant cerebral atrophy and its adverse effect on nervous as well as visual systems. Lethal dose of methanol vary from 30 to 240 ml equivalent to 1gr/kg (1/2ml/kg). Although 30 ml of 40% solution is considered as the minimum lethal dose, however, reports of survival following ingestion of 500 to 600 ml have been reported (8).

Excessive consumption of alcohol produces larger amounts of formic acid beyond the capability of the enzyme to convert it to CO2, increased synthesis of formic and its accumulation lead to lactic acidosis and inhibition of unitcochron respiration (9, 10). 

In a report from the American Association of Poison Control Centers (AAPCC). 44 out of 979 methanol poisoning victims had major complications. Four of these cases succumbed to death (11). A similar neurological complications including bilateral putamen necrosis, diffuse white matter necrosis, topical lesions in the cerebellum and hypothalamus have been reported by Taheri et al. (1). Similarly, Jain et al. reported bilateral putamen necrosis and cerebral hemorrhage associated with white matter lesions in the brain and cerebellum in patients poisoned with methanol asserted (12). 

It has been shown that delay referral is associated with cerebral sequels, visual impairment, Parkinson's syndrome, paraplegia and polyneuropathy (13). 

Currently, physicians of ICU wards try to remove patients from the mechanical ventilators as early as possible. Persistent period of ventilation as observed in this study is of particular concern. Because irrespective to economical burden, prolonged period of ventilation leads to ventilator dependency and more severe-related complications (4). Currently, the first attempts in removing from mechanical ventilation is usually unsuccessful in about 20% of patients in ICU (5, 14). 

This issue is usually attributed to imbalance between the respiratory muscle strength and its capacity (5). This was illustrated in a study of Parkinson’s disease by Hass et al. In these patients, the mouth/breath pressure which correlates with respiratory muscle strength is lower than expected (15). In the present study, also the difficulties in weaning patients from the ventilator should be attributed to respiratory muscle weakness due to toxic effects of methanol or its metabolites as well as to persistent acidosis. Treatment of this patient began 30 hours after ingestion due to delayed clinical presentation. This issue should be explained by concomitant ingestion of both methanol and ethanol which is present in homemade alcohol.

 Because during illegal distillation of homemade alcohol both ethanol and methanol are expected to be produced. Ethanol consumption, through its inhibitory effect on alcohol dehydrogenase results in slower conversion of methanol to more toxic metabolites and so increasing to toxic metabolite level requires greater time. 

**Conclusion:** This study indicates a case of methanol poisoning by ingestion of illegally produced homemade alcohol resulting in major neurological complications such as cerebral bleeding and atrophy and related motor disability as well as irreversible vision loss.
